# Association between the disease severity and quality of life of patients with psoriasis in a tertiary government hospital in the Philippines: A retrospective cross-sectional study

**DOI:** 10.1016/j.jdin.2023.12.014

**Published:** 2024-04-07

**Authors:** Arbie Sofia P. Merilleno, Francisco D. Rivera, Maria Sharlene P. Temblique

**Affiliations:** a Department of Dermatology, Rizal Medical Center, Pasig, Philippines; b Research and Innovation Institute and Department of Medicine, Women's College Hospital, Toronto, Ontario, Canada; c Division of Dermatology, Department of Medicine, University of Toronto, Toronto, Ontario, Canada

**Keywords:** comorbidities, disease severity, DLQI, PASI, psoriasis, quality of life

*To the Editor:* Psoriasis results to physical, psychological, social, and economic impact to patients. Information on the disease severity, demographics, and clinical characteristics of patients has a bearing on the behavior, prognosis, and treatment of the disease. An analytical cross-sectional study was conducted involving patients with psoriasis in Rizal Medical Center, a tertiary government hospital in the Philippines. Psoriasis Area and Severity Index, Dermatology Life Quality Index (DLQI), and demographic and clinical characteristics of patients were obtained to identify predictor variables of PASI and DLQI.

The study included 155 patients with psoriasis using a total enumeration sampling technique. A total of 57.4% had moderate to severe disease, and 63.9% concluded that the disease had very large to extremely large effect on their quality of life. Psoriatic arthritis worsened the severity of the disease (Table I). In contrast, age, presence of comorbidities such as hypertension, dyslipidemia, cardiovascular disease, depression, family history, and psoriatic arthritis worsened the quality of life of patients (Table II).

**Table I:** Association of the patient demographic and clinical characteristics with disease severity among patients with psoriasis

Patient characteristics	Total (*n* = 155)	Mild (PASI score of <10) (*n* = 66)	Moderate to severe (PASI score of ≥ 10) (*n* = 89)	*P* value
Demographic variables				
Age, y				
19-30	61	31 (50.8)	30 (49.2)	.878
31-40	39	13 (33.3)	26 (66.7)	.177
41-50	24	8 (33.3)	16 (66.7)	.319
51-60	19	8 (42.1)	11 (57.9)	.964
≥ 60	12	6 (50.0)	6 (50.0)	.588
Sex				
Female	89	40 (44.9)	49 (55.1)	
Male	66	26 (39.4)	40 (60.6)	.490
Region/residence				
National Capital Region (NCR)	87	38 (45.2)	49 (56.3)	.652
Non-NCR	68	28 (40.6)	40 (58.8)	
Marital status				
Single	91	42 (46.2)	49 (53.9)	.283
Married	61	23 (37.7)	38 (62.3)	.323
Widow/er	3	1 (33.3)	2 (66.7)	.744
Clinical profile variables				
Duration of disease				
Early onset (<40 y/o)	93	37 (39.8)	56 (60.2)	.817
Late onset (≥ 40 y/o)	26	11 (42.3)	15 (57.7)	.817
Aggravating factors				
Stress	118	52 (45.2)	66 (54.8)	.504
Infection	27	10 (37.0)	17 (63.0)	.521
Skin trauma/sunburn	25	11 (44.0)	14 (56.0)	.875
Diet	20	7 (35.0)	13 (65.0)	.463
Others	19	10 (52.6)	9 (47.4)	.344
Alcohol drinking	17	6 (35.3)	11 (64.7)	.520
Smoking	11	5 (45.5)	6 (54.6)	.841
Medications	9	2 (22.2)	7 (77.8)	.203
Inflammatory bowel disease	1	0	1 (100.0)	.388
Presence of comorbidities				
Hypertension	29	13 (44.8)	16 (55.2)	.786
Diabetes	22	9 (40.9)	13 (59.1)	.864
Arthritis	20	5 (25.0)	15 (75.0)	.088
Others	16	6 (37.5)	10 (62.5)	.920
Dyslipidemia	14	7 (50.0)	7 (50.0)	.556
Tuberculosis	11	4 (36.4)	7 (63.6)	.665
Depression	8	4 (50.0)	4 (50.0)	.663
Cardiovascular disease/stroke	7	4 (57.1)	3 (42.9)	.425
Family history of psoriasis	71	28 (39.4)	43 (60.6)	.467
Psoriatic arthritis/joint involvement	24	3 (12.5)	21 (87.5)	.001
Nail involvement	106	43 (40.6)	63 (59.43)	.456
Treatment received				
Topical therapy	126	52 (41.3)	74 (58.7)	.491
Systemic therapy	68	26 (38.2)	42 (61.8)	.333
Phototherapy	26	9 (34.6)	17 (65.4)	.369

Statistical test used: Pearson chi-square test.

**Table II:** Association of the patient demographic and clinical characteristics with quality of life among patients with psoriasis

Disease severity	Total (*n* = 155)	Effect on patient’s quality of life			*P* value
		No effect (0-1) (*n* = 6)	Small to moderate effect (2-10) (*n* = 50)	Very large to extremely large effect (≥11) (*n* = 99)	
Demographic variables					
Age, y					
19-30	61	2 (3.3)	22 (36.1)	37 (60.7)	.015
31-40	39	0	9 (23.1)	22 (76.9)	.173
41-50	24	2 (8.3)	8 (33.3)	16 (58.3)	.507
51-60	19	2 (10.5)	6 (31.6)	10 (57.9)	<.001
≥60	12	0	5 (41.7)	7 (58.3)	.516
Sex					
Female	89	3 (3.4)	30 (33.7)	56 (62.9)	.860
Male	66	3 (4.5)	20 (30.3)	43 (65.2)	
Region/residence					
National Capital Region (NCR)	87	3 (5.4)	19 (21.8)	65 (74.7)	.247
Non-NCR	68	3 (3.4)	31 (45.6)	34 (50.7)	
Marital status					
Single	91	1 (1.6)	18 (29.5)	42 (68.9)	.561
Married	61	5 (4.9)	31 (34.1)	55 (60.4)	.605
Widow/er	3	0 (0.0)	1 (33.3)	2 (68.7)	.897
Clinical profile variables					
Duration of disease					
Early onset (<40 y/o)	93	5 (5.4)	40 (43.0)	84 (90.3)	.814
Late onset (≥ 40 y/o)	26	1 (3.8)	10 (24.4)	15 (57.7)	
Aggravating factors					
Stress	118	6 (5.1)	46 (39.0)	66 (55.9)	.771
Infection	27	1 (3.7)	9 (33.3)	17 (63.0)	.796
Skin trauma/sunburn	25	1 (4.0)	10 (40.0)	14 (56.0)	.932
Diet	20	1 (5.0)	6 (30.0)	13 (65.0)	.752
Others	19	2 (10.5)	8 (42.1)	9 (47.4)	.424
Alcohol drinking	17	0	6 (35.3)	11 (63.7)	.553
Smoking	11	0	5 (45.5)	6 (54.5)	.660
Medications	9	0	2 (22.2)	7 (77.8)	.412
Inflammatory bowel disease	1	0	0	1 (100.0)	.689
Presence of comorbidities					
Hypertension	29	5 (17.2)	8 (27.6)	16 (55.2)	.004
Diabetes	22	3 (3.8)	6 (27.3)	13 (59.1)	.117
Arthritis	20	2 (10.0)	3 (15.0)	15 (75.0)	.068
Others	16	0	7 (43.8)	9 (10.1)	.572
Dyslipidemia	14	3 (21.4)	4 (28.6)	7 (50.0)	.015
Tuberculosis	11	2 (18.2)	2 (18.2)	7 (63.6)	.077
Depression	8	2 (25.0)	2 (25.0)	4 (50.0)	.032
Cardiovascular disease/stroke	7	3 (42.9)	1 (14.3)	3 (42.9)	<.001
Family history of psoriasis	71	7 (18.9)	27 (38.0)	37 (52.1)	<.001
Psoriatic arthritis/joint involvement	24	1 (4.2)	2 (8.3)	21 (87.5)	.004
Nail involvement	106	7 (6.7)	35 (33.3)	63 (60.0)	.338
Treatment received					
Topical therapy	126	5 (4.0)	47 (37.3)	74 (58.7)	.357
Systemic therapy	68	2 (2.9)	24 (35.3)	42 (61.8)	.427
Phototherapy	26	1 (4.0)	7 (28.0)	17 (68.0)	.565

Statistical test used: Pearson χ^2^ test.

Results showed that joint involvement has a strong association with disease severity. This can be supported by studies that suggest that patients with high skin severity are twice more likely to have increased joint involvement.^1^ Patients with joint and nail involvement also had higher DLQI scores because psoriatic arthritis could contribute to disability or physical limitation. The visible disfigurement caused by psoriasis heavily impacts the emotional and psychological well-being of patients. In this study, ages 19 to 30 years and ages 51 to 60 years were significantly correlated to lower quality of life. Psoriasis usually causes body image dissatisfaction and lower self-esteem especially for younger patients, which is highlighted by social media’s beauty standards.^2^ Meanwhile, for elderlies, they usually fear that the disease is transmissible, so they stay away from their families and friends, and limit their social interactions, causing mental health issues and worsening the quality of life.^3^ Moreover, quality of life and presence of comorbidities, such as hypertension, dyslipidemia, cardiovascular disease, and depression were highly correlated. These diseases aggravate the burden of the patient because it negatively impacts daily activities of the patient. Financial burden of patients is substantial^4^ and to experience other comorbidities brings additional threat to the financial and social status of patients. Quality of life was also affected by the presence of family history of psoriasis. This is likely due to patient’s self-perception on the distress and burden experienced by close relatives. Psoriasis affects not only the quality of life of patients themselves, but also the quality of life of their family members.^5^ To experience this negative impact on their well-being, it increases patient’s fear and psychological distress.^5^

Psoriasis affects the well-being of a patient. Quality of life was affected by the severity of the disease and different demographic and clinical factors of a patient. It is recommended that clinicians take a holistic approach when managing patients with psoriasis and address their needs to improve their quality of life. These patients should be referred to other specialties, which can manage their comorbid conditions which further aggravate their quality of life.

## Conflicts of interest

None disclosed.
